# Participatory Development and Pilot Testing of an Adolescent Health Promotion Chatbot

**DOI:** 10.3389/fpubh.2021.724779

**Published:** 2021-11-11

**Authors:** Laura Maenhout, Carmen Peuters, Greet Cardon, Sofie Compernolle, Geert Crombez, Ann DeSmet

**Affiliations:** ^1^Department of Movement and Sports Sciences, Ghent University, Ghent, Belgium; ^2^Department of Experimental-Clinical and Health Psychology, Ghent University, Ghent, Belgium; ^3^Research Foundation Flanders (FWO), Brussels, Belgium; ^4^Faculty of Psychology and Educational Sciences, Université Libre de Bruxelles, Brussels, Belgium; ^5^Department of Communication Studies, Faculty of Social Sciences, University of Antwerp, Antwerp, Belgium

**Keywords:** chatbot, development, person-based approach, adolescents, health promotion

## Abstract

**Background:** The use of chatbots may increase engagement with digital behavior change interventions in youth by providing human-like interaction. Following a Person-Based Approach (PBA), integrating user preferences in digital tool development is crucial for engagement, whereas information on youth preferences for health chatbots is currently limited.

**Objective:** The aim of this study was to gain an in-depth understanding of adolescents' expectations and preferences for health chatbots and describe the systematic development of a health promotion chatbot.

**Methods:** Three studies in three different stages of PBA were conducted: (1) a qualitative focus group study (*n* = 36), (2) log data analysis during pretesting (*n* = 6), and (3) a mixed-method pilot testing (*n* = 73).

**Results:** Confidentiality, connection to youth culture, and preferences when referring to other sources were important aspects for youth in chatbots. Youth also wanted a chatbot to provide small talk and broader support (e.g., technical support with the tool) rather than specifically in relation to health behaviors. Despite the meticulous approach of PBA, user engagement with the developed chatbot was modest.

**Conclusion:** This study highlights that conducting formative research at different stages is an added value and that adolescents have different chatbot preferences than adults. Further improvement to build an engaging chatbot for youth may stem from using living databases.

## Introduction

Insufficient physical activity and sleep, too much sitting time and an unhealthy diet contribute to the development of overweight, obesity, non-communicable diseases (including for example diabetes, cardiovascular diseases, certain types of cancer), and mental health problems (1–5). Evidence shows that health behaviors track from childhood into adulthood (6–8), and that health behaviors and mental health often deteriorate in adolescence (9). In Flanders (i.e., Dutch-speaking part of Belgium), 83.5% of adolescents aged 11–17 years do not meet national guidelines for physical activity (10), half of 11–15 year old adolescents do not meet the norm of 8-h of sleep (11), more than 90% is spending too much time (>2 h/day) on screen-related sedentary behavior (12), and around half do not take breakfast daily (13). Therefore, early adolescence is a crucial period to focus on the prevention of health problems (14). Mobile and computer devices are increasingly used to deliver health promotion interventions (15, 16). Despite the interest of adolescents for digital health content (9), youth's adherence to and engagement with digital health interventions is rather low (17–21). This may be problematic as user engagement is considered crucial for intervention effectiveness (22). One potential reason for low adherence and user engagement is the lack of human interaction in digital health interventions (18, 23–27). A chatbot that provides a human-like interaction may overcome these problems (19, 23, 28–33). Chatbots are computer programs designed to mimic human conversations through text (28, 34–38). The few available studies on chatbots in health promotion have shown that these can increase user engagement with digital health interventions (28) and might be effective in improving healthy lifestyles and mental health outcomes (19, 25, 39, 40).

Engagement with digital interventions includes both (1) the extent (e.g., amount, frequency, duration, depth) of usage and (2) the subjective experience characterized by attention, interest and affect (41). Interventions may be more engaging when user preferences and needs are integrated in the development process (42–44). Current chatbot literature has mainly studied user experience (33, 37, 45–52), the effect of chatbots on certain (health) outcomes (19, 25, 39, 40), important characteristics to include (44, 53–55) or challenges to overcome (34, 35, 56, 57). Literature on how chatbots are developed based on user preferences and needs is largely lacking. Some studies did include the perspective of end users, but only two focused on health (58–63) and very few on youth (51, 64–66). The studies of Crutzen et al. (51) and Gabrielli et al. (65) explored adolescent views on a prototype chatbot. However, chatbot preferences before the testing of a pre-developed prototype were not explored. Beaudry et al. (66) organized co-creative workshops with adolescents with chronic conditions, but only reported the satisfaction with the workshops and the initial chatbot testing (e.g., on usability of the technology, engagement rate, and user behavior), and not the content or output of the co-creation sessions. In all three studies, it is unclear how adolescents' perspectives have been integrated into the initial phases of the chatbot development. Given the lack of research on the participatory development of youth health promotion chatbots and their potential to increase engagement with digital health interventions, this study aims to explore what youth expect from and prefer in health promotion chatbots. These expectations and preferences can provide directions to the development and use of chatbots for youth health promotion purposes.

## Theoretical Rationale

### Person-Based Approach

**Figure 1:**
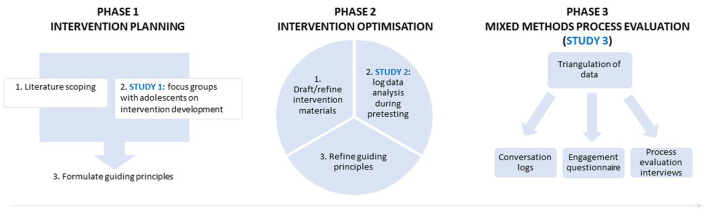
Overview of the chatbot development phases, based on the PBA to intervention development (68, 69).

To ensure that the needs and perspectives of the target end-users are embedded in a health promotion chatbot, the “person-based approach” (PBA) was used as theoretical framework to guide this development process. PBA is a stepwise process that can be divided into three stages: (1) intervention planning, (2) intervention optimization, and (3) mixed-methods process evaluation (see Figure 1) (67–69). The fundamental aim of PBA is to build iterative in-depth qualitative research into the entire development process to ensure that the intervention fits with the psychosocial context of the end-users (69–71). This approach has been used for other digital applications such as health promotion and illness self-management (71) but has not yet been applied to the development of a youth health promotion chatbot.

This paper describes the research during the person-based development process of an adolescent health promotion chatbot. The chatbot was integrated within a digital intervention which contains three components: (1) a self-regulation app with associated Fitbit for goal setting, monitoring and feedback, (2) a video narrative (e.g., adolescents could access a short video of a youth series in the app every week) and (3) a virtual coach (“chatbot”). Screenshots of the app are included in Supplementary Material 1. The integration of a chatbot into the self-regulation app aimed to increase user engagement by offering adolescents social support. The intervention was focused on achieving sufficient sleep and physical activity, increasing daily breakfast intake and reducing sedentary behavior to promote the mental well-being of adolescents (between 12 and 15 years of age) (3, 5, 72–74). Of note, the chatbot was focused on primary prevention and not on treatment of mental health problems. When signs of mental disorders were detected in the questions users asked the chatbot, the user was referred to appropriate websites of professional organizations.

## Methods and Results

Three successive studies were performed to answer the following research questions (RQ): (1) What are style preferences of adolescents (for both features and content) of a chatbot?, (2) Which health promotion-related questions do adolescents ask a chatbot?, (3) Which answers do they expect from the chatbot?, (4) Does the developed chatbot work as expected in a real-life setting?, and (5) How engaged are adolescents with the chatbot? These research questions were addressed through three studies in three different stages of the theoretical framework: (1) a qualitative focus group study, conducted in phase 1 on Intervention Planning (RQ1, RQ2, RQ3), (2) a log data analysis during pretesting, conducted in phase 2 on Intervention Optimization (RQ2, RQ4), and (3) a mixed-method pilot testing of the developed chatbot, conducted in phase 3 (RQ2, RQ4, RQ5). Figure 1 shows how the findings of each study were integrated in the development process of the chatbot, following PBA.

### Ethics Approval and Consent to Participate

All research has been approved by the Committee of Medical Ethics of the Ghent University Hospital (Belgian registration number: EC/2019/0245) and the Ethics Committee of the Faculty of Psychology and Educational Sciences of Ghent University (registration number: 2019/93). Written informed consent from the participants and their parents was obtained prior to participation in the different studies.

### Participants

The target population for all studies were adolescents between 12 and 15 years old. Participants were included if they: (1) were in the 7th, 8th, or 9th grade (1st−3rd year of secondary school), and (2) had a good understanding of Dutch. Exclusion criteria were schools of special education and education for non-native speakers (in preparation for regular education).

#### Phase 1: Intervention Planning

This phase aimed to establish the features (i.e., design elements, chatbot characteristics, and software settings (e.g., programmed language use)) and content (i.e., questions and answers) of the chatbot that youth consider to be important.

It included: (1) a scope of existing literature for chatbot features and (2) a qualitative study into user preferences on questions, answers, and preferred features for health promotion chatbots. The insights from this phase provided: (3) the “guiding principles” for intervention development, in the PBA. These principles direct the entire development process: they describe the core intervention objectives and features needed to achieve these objectives (67–69). To clarify, the ultimate goal (or program objective) of the intervention is to improve healthy lifestyle behaviors. The core intervention objectives reflect the change that is needed to reach that ultimate goal, and could be considered as “mechanisms of change” or also as change objectives when drawing the parallel with the Intervention Mapping Protocol. The overall intervention, that comprises the app, chatbot and narrative, is based on the Health Action Process Approach (HAPA) (75). Within the self-regulation app, adolescents can set goals (action planning), come up with solutions to possible barriers (coping planning), self-monitor their behavior with a Fitbit and gain rewards using coins that buys them accessories for their avatar. To also address the “pre-intenders” or motivational phase within the HAPA-model, the Elaboration Likelihood Model (76) was used in creating a youth series that could motivate adolescents for behavior change. The HAPA model also stresses the importance of social support. The chatbot was envisaged as a complementary tool for social support to the self-regulation app, that is mainly an individual intervention. The guiding principles provide an overview of the ways in which the chatbot will help support behavior change and maximize engagement (67, 68), in addition to the other two intervention components (app, narrative) that are not further discussed in this paper.

##### Literature Scoping

A narrative, non-exhaustive literature search revealed several important chatbot features for the general population (note: these may include youth, as the age range was not always provided in these publications).

According to the Computers Are Social Actors (CASA) paradigm (77, 78) humans exhibit social reactions that are similar to those observed in interpersonal communication when interacting with computers. More specifically, humans automatically apply social rules, expectations, and scripts known from interpersonal communication and apply it to the computer (38). In this regard, researchers agree that when developing a chatbot, attention should go both to technical and social aspects (38). Feine et al. (38) recently developed a taxonomy of social cues for conversational agents. Social cues were divided into four major categories (i.e., verbal, visual, auditory, and invisible) and ten subcategories. Based on this taxonomy, we searched the literature for examples of social cues, first for the general population (of which adolescents are a part), then specifically for adolescents. Since this project developed a text-based chatbot, and not a voice-based conversational agent or embodied conversational agent (ECA) that use both verbal and non-verbal communication, no further information was sought within the “auditory” category. Within the verbal category, important features for the general population included the use of humor and empathy, engaging in small talk, appropriate referrals in case of safety-critical health issues, expressing a name, and overall chatbot description in creating certain user expectations, short and precise interaction and a variation in system responses and dialogue structures (e.g., not giving the same response to the same question each time). Moreover, within the visual category, the agent appearance also seems important, by giving a pleasant profile picture (44, 53–57, 79). More specifically for adolescents, within the verbal category they prefer the use of empathy and anonymity, the possibility to engage in small talk, asking questions related to topics that are difficult to talk about with their parents, free dialogue (i.e., unconstrained language input) and the avoidance of redundant answers. Within the visual category they prefer a chatbot with a personality (e.g., like a nice, smart old friend, someone you can trust). Finally, within the invisible category, and more specifically the subcategory “chronemics” (i.e., the role of time and timing in communication) fast responses seem to be important (51, 64, 65). The social cues taken into account in the further development process of this chatbot can be found in Supplementary Material 2.

##### Study 1: Focus Groups With Adolescents on Intervention Development

The aim of the focus groups was to gain insights in: (1) preferences of both content and design, (2) which questions adolescents would ask a chatbot, and (3) which answers they would expect.

*Interview Guide Design*. Chatbots need a basic input set or database that is further completed during the process of using the chatbot. To get acquainted with the type of questions youth ask in relation to health, the researchers consulted a list of 319 anonymized chat threads from an online youth helpline. An ethics agreement form was signed between the helpline and researchers, requiring that no verbatim responses that can be traced back to a specific adolescent would be used in the development or in any communication. The list included chat threads on physical activity, breakfast, sedentary behavior, sleep and mental health. These 319 chat threads helped to form the initial database, and also to create the interview guide and probing material for the focus groups, by understanding the language adolescents commonly use when talking about (mental) health and finding the most frequently asked questions and answers. More detail on the chat threads themselves can be found in Supplementary Material 3. The interview guide can be found in Supplementary Material 4.

*Participants*. Flemish secondary schools were selected via convenience sampling. Forty-five schools were contacted of which four schools agreed to participate (i.e., response rate of 9%). Reasons for non-participation in the other schools were lack of interest and lack of time. In three of the participating schools the chatbot was discussed. The fourth school participated in focus groups on the development of the other intervention components (app, narrative). In selecting the three schools, attention was paid to having a good mix of general academic (three focus groups) and technical-vocational education (three focus groups). Six focus groups were conducted with 4–7 participants each. Some classes were small and in these classes, all consenting pupils participated in the focus groups. In bigger classes, a selection of consenting pupils participated in these chatbot focus groups while other pupils participated in focus groups on other intervention components.

*Procedure*. The focus groups were conducted in May 2019 and took place at school during one class hour. At the start, study information was provided, confidentiality of the discussions was emphasized, participants' questions were answered and informed consent forms were collected from adolescents and their parents (distributed by the teachers a week before). Demographic information was collected in a self-report questionnaire. Each focus group started with a couple of warm-up questions, including information on what a chatbot is, showing an online example^1^; and asking whether they had used a chatbot before and would use it in the future (e.g., intention to use). Participants received a cinema ticket as incentive.

*Analysis*. The focus group discussions were audiotaped. Audio-recordings were transcribed verbatim and coded via NVivo 12.0 software using inductive thematic analysis. LM read the transcripts and developed categories of responses. A sample of 10% of responses was independently double-coded by another member of the research team (ADS). An intraclass correlation coefficient (ICC) of 0.70 was obtained, which can be considered good.

***Results** Sample description*. Thirty-six adolescents aged between 12 and 15 years participated, among whom 29 girls and 7 boys. Very few Flemish adolescents had used a chatbot before (e.g., Siri and Warm William), but most expressed to be willing to use a chatbot on the condition that it is well-designed.

*Findings*. The results of the focus groups can be categorized into three main themes: (1) style preferences divided into (1a) content preferences and (1b) design preferences, (2) findings regarding questions adolescents would ask, and (3) answers they would expect. Illustrative quotes can be found in Tables 1–**3**.

**Table 1:** Results from the focus groups regarding style preferences.

**Theme**	**Subtheme**	**Quote**
**Style preferences: Content**	Unconstrained language input	[About Warm William] “*I did not like he always asked the questions and you had to answer, so you couldn't ask anything yourself.” (FG 1, grade 7, general academic track)*
	Be clear about chatbot capabilities	“*I think that before you use the chatbot, you should already know like: I'm not going to start joking here. Because with Siri everyone does that, but it's not clear that that's not the intention or something, yeah…” (FG 2, grade 9, general academic track)*
	Not childish	[About Warm William] “*I thought that was good for kids, just the way he spoke and with the profile picture of a blue bear.” (FG 1, grade 7, general academic track)*
	Empathy	“*(He has to) be a little bit concerned with you” (FG 1, grade 7, general academic track)*
	Humor	“*Or if the robot's joking, that'd be a laugh.” (FG 2, grade 9, general academic track)*
	Non-judgmental	“*I think that it is something easy to go to. Because it's actually quite a neutral opinion and it's nobody I know… Yeah, it doesn't have an opinion about things…” (FG 3, grade 9, general academic track)*
	Trustworthy	“*That it gives you the feeling that you are safe there and that it never spreads something or that things really stay there and that no one else is ever going to know.” (FG 2, grade 9, general academic track)*
	Personality	“*That it has such a personality, a name, etc. Yes, and if you get to know the chatbot like that, you will feel better about it.” (FG 2, grade 9, general academic track)*
	Ability to follow the conversation	“*And he has to go along with the conversation. Yeah, so don't suddenly start talking about anything else.” (FG 1, grade 7, general academic track)*
	Ability to memorize previous conversations	“*But, like in Messenger, you can keep seeing that conversation, too. So that somebody can say 'yes, I've listened to your advice and, um, this helped and this didn't' and then the chatbot can say 'yes, try that again' or something.” (FG 2, grade 9, general academic track)*
	Language use (i.e., youth language)	“*Yeah, I think it sounds more fun when it's in youth language. That you really feel like 'someone my age' and so on.” (FG 2, grade 9, general academic track)*
	Emoji's	*Don't overdo it with emojis, but use one now and then… (FG 2, grade 9, general academic track)*
	Notifications	“*Yeah, that he asks you something, but not all the time the <ping ping ping sound>. That you don't get messages like that all the time.” “Just once a day or so, around 4 p.m.” (FG 1, grade 7, general academic track)*
	Tailoring	“*I think the answers should be a bit personal, not in the way of a robot that gives standard answers. For example, if you don't like jogging at all, but you like racket sports or something like that, the chatbot must look for a solution or give tips in this field. Instead of making lost efforts to encourage you to go jogging.” (FG 3, grade 9, general academic track)*
**Style preferences: Design**	Like message apps known to adolescents	“*But, maybe just a little bit like a message app, like when you get a message from somebody, that it looks a little bit the same. Also with, yeah… that you can open it directly and there's a face of, um, yeah… maybe more like Messenger.” (FG 3, grade 9, general academic track)*
	Cheerful design	*Interviewer: “What do you think the chatbot should look like?”* *Adolescent: “Cheerful.”* *Interviewer: “Cheerful, and what exactly is cheerful to you?”* *Adolescent: “Just with different colors etc.” (FG 6, grade 9, vocational track)*
	Ability to personalize (e.g., colors, backgrounds, and profile picture)	“*Like with WhatsApp you can change the background of the chat itself. Or like that you can turn on dark-theme in Messenger or something like that.” (FG 3, grade 9, general academic track)*
	Ability for the user to delete the conversation	“*That you can also choose to ‘delete the chat' or something like that. Because otherwise you have the problem of privacy again. If someone accidentally opens your computer or your mobile phone and the conversation is still there, then they will see all your problems” (FG 2, grade 9, general academic track)*

Within the theme of (1a) content preferences, the following sub-themes emerged: the chatbot should have an unconstrained language input; it should be clear what its capabilities and limits are; it should not be childish but instead be empathic, humoristic, non-judgmental, and trustworthy. Adolescents thought it would be of value if the chatbot had a personality and the ability to follow the conversation or memorize previous conversations. They also would like a tailored chatbot that used youth language with emoji's and could send (not too many) notifications. In terms of (1b) design preferences, they would like to have a chatbot with a design similar to message apps that they are already familiar with, a cheerful design with the possibility of personalization or for the user to be able to delete the conversation (Table 1).

Regarding the questions adolescents would ask the chatbot (subtheme 2), they reported they would mainly ask small talk questions, questions that they do not dare or cannot ask their parents or friends, and also very broad questions that do not immediately fit in with the chatbot's purpose (e.g., study tips, love issues, etc.) (Table 2).

**Table 2:** Results from the focus groups regarding questions adolescents would ask.

**Theme**	**Subtheme**	**Quote**
**Questions**	Small talk	“*I would ask questions like how old are you, or what have you already done today?” (FG 6, grade 9, vocational track)*
	Questions difficult to ask parents	“*I'd rather ask for something where you need someone else's opinion. For example, things that aren't really clear when your parents explain it. Or things that you don't want to ask your parents*.” *(FG 3, grade 9, general academic track)*
	Broader than the purpose of the chatbot	“*I would ask all my questions about, yes, my body or about movement and, yes, all kinds of things… not just about that specific topic (e.g., health behaviors), but also about other things.” (FG 2, grade 9, general academic track)*

Lastly, the adolescents indicated that the chatbot answers (subtheme 3) should be formulated accurately, realistically and in a positive way. There were both positive and negative opinions regarding referral to other sources, parents, and websites. If referrals are made to websites, the link should be immediately clear (Table 3).

**Table 3:** Results from the focus groups regarding answers adolescents would expect.

**Theme**	**Subtheme**	**Quote**
**Answers**	Accurate answers	“*But if it doesn't give you the answers you need, I'm not going to use the chatbot…” (FG 1, grade 7, general academic track)*
	Realistic answers	“*For example, that there are logical answers, like the answer you should drink one hundred liters of water per day, you know that is not right, answers that really help you are better.” (FG 1, grade 7, general academic track)*
	Formulated in a positive manner	“*Certainly not oblige, but give more advice, because otherwise it's like a parent and that's exactly the reason they wouldn't tell their parents.” (FG 3, grade 9, general academic track)*
	Negative opinion about referring to other resources	“*But, I don't think he should refer you like that, otherwise you'd look it up yourself.” (FG 1, grade 7, general academic track)*
	Positive opinion about referring to other resources	“*They [health care providers] are more alive or something than that robot, they have feelings.” (FG 4, grade 9, vocational track)*
	Negative opinion about referring to parents	“*I ask the chatbot something but he just refers you directly, then yes, if you don't want to say it to your parents, then you sit there, because then I wouldn't say it to my parents and I wouldn't send anything to the chatbot anymore, because then he just hasn't helped you.” (FG 1, grade 7, general academic track)*
	Positive opinion about referring to parents	“*But I do think when you say to that robot 'I'm suicidal' that he must say you should go to your parents or something.” (FG 1, grade 7, general academic track)*
	Negative opinion about referring to website	“*If I would get that, I wouldn't go to those sites and I wouldn't send him again either, so I would just find that my problem hasn't been solved yet. I certainly wouldn't go to those sites.” (FG 1, grade 7, general academic track)*
	Positive opinion about referring to website	“*I wouldn't mind, you will know that the advice is not just from the chatbot, but then you can also see from other websites that it's also a good advice… then you have two sources you can compare and if that matches then that gives a feeling of 'this is right'.” (FG 2, grade 9, general academic track)*
	Referring to website link should be clear	“*If the link is there and all you have to do is press the link, I think I would. But not when you go to a website and you first have to search… You should really immediately find what you need: 'Tips about sleeping'. And that you don't have to search for that.” (FG 2, grade 9, general academic track)*

Supplementary Material 5 shows the social cues that emerged from these focus groups and that were added to the cues found in literature.

##### Formulate Guiding Principles

As a last step within the first phase of intervention planning, guiding principles were formulated (68, 69, 71). They are based on the results of the focus groups. Table 4 shows the guiding principles and how they are incorporated into the prototype.

**Table 4:** Guiding principles and examples of how these were integrated in the chatbot prototype.

**Core intervention objectives**	**Key features needed to achieve these objectives**	**Examples how this was included in the chatbot**
Provide social support	• Unconstrained language input (i.e., free dialogue) • Use youth language • Give the chatbot a “human-like” look (e.g., empathic and humoristic) • Accurate answers • Realistic answers	• Using the software platform Dialogflow, the adolescents are free to ask any question they want to the chatbot, the conversation starts from the adolescent him- or herself. • Adapting language style (e.g., no scientific words, not too formal or too mature but simple and concise answers), splitting long pieces of text into shorter ones, using emoji's and adding “life quotes” to some chatbot responses. • Within the answers of the chatbot, care was taken to be empathic (“*I understand what you mean,” “that must be annoying,” “that sounds like fun”*) and to use some humor by adding jokes to the input. • Clustering the chatbot input according to certain health domains. A cluster consists of all kinds of different questions to which one joint answer could be given (see section Draft/Refine Intervention Materials). By having different training phrases within a cluster, there was less chance of the chatbot giving a wrong answer to a question. • The formulated chatbot answers were sent to stakeholders and experts within the theme for feedback.
Engagement	• Small talk • Attractive design with personalization options • Notifications	• Integrate standard small talk (e.g., exchanging greetings, how are you, who are you, how old are you, etc.). • Design in Messenger or WhatsApp style, for example when the chatbot is “still typing,” an image with three dots appears. • A settings page was created where users have the option to change the background of the chat, change the font color and the option to delete the entire conversation. • The chatbot weekly sends two messages to the adolescents (after school) so that they are drawn back to the app. One message motivates adolescents to set goals in the self-regulation app, the other gives the tip that a new episode of the narrative series is uploaded in the app.
Provide knowledge	• Giving tips • Referring to websites with the right information	• Website links were only added for extra information, but the chatbot answered the question itself as much as possible. This way, adolescents would have the choice if they wanted to read extra information on a website.
Act as a guide	• Referring to the appropriate organizations which can provide proper help	• Referrals to other resources were suggested only when really needed on the basis of frequently used terms which suggest mental difficulties (e.g., suicide, depression, self-mutilation, physical complaints, etc.).

#### Phase 2: Intervention Optimization

In the second stage, i.e., the intervention optimization stage (67, 68), a prototype of the chatbot was developed based on the guiding principles and was pretested by the target users. Dialogflow^2^ is a platform that allows free dialogue and was used as the software platform for this chatbot. The conversation logs of the pre-testers were closely monitored and used to refine the guiding principles (68) and further fine-tune the intervention (see Figure 1).

##### Draft/Refine Intervention Materials

To meet content and design preferences, the chatbot included free dialogue, used youth language, was given a human-like look using empathy and humor, gave accurate and realistic answers, included small talk, got an attractive design, provided notifications, and gave tips and referrals to websites or other resources (Table 4). The collected chatbot input, based on the chat threads and focus groups, was clustered in certain health domains. A cluster consists of all different questions to which one joint answer could be given [e.g., specific questions about how many hours of television, mobile phone, social media, computer, gaming, Netflix is healthy/unhealthy; comments about not liking to do anything else but play games/watch television; or comments about spending a lot of screen-related time (via different devices) were all categorized in one cluster “screen-related sedentary behavior”]. Some preferences, such as tailoring to their specific needs and interests, and the ability to ask the chatbot questions broader than the chatbot purpose, could not be implemented within the time frame of this development study. A screenshot from the prototype (in Dutch) can be found in Supplementary Material 6.

##### Study 2: Log Data Analysis During Pretesting

Once the prototype was drafted, the second step within this phase consisted of the pretesting of the prototype. The chatbot prototype consisted of a simplified website format where the adolescents only had the option to type in a question. Pretesting was on just one health domain (i.e., sleep) for the sake of efficiency. The pretesting assessed whether content and technical aspects functioned properly. Results from testing one health domain could therefore also be extrapolated to the other topics in the prototype.

*Participants*. A convenience sample of adolescents was recruited via the personal network of the researchers. Information about the study was provided via e-mail. Interested adolescents were asked to provide both adolescent and parental informed consent and received information on how to install the chatbot. A total of 17 adolescents between 12 and 15 years were contacted, including 6 boys and 11 girls. Six girls participated.

*Procedure*. Adolescents were invited to ask the chatbot questions about the theme “sleep” for 1 week. The conversation logs showed (1) which questions adolescents asked and (2) how the chatbot responded to this.

*Results*. The log data resulted in a list of all questions asked by adolescents to the prototype chatbot, which was then compared with the database based on the input from the chat threads and focus groups (phase 1). We checked which questions already fit into the existing answer clusters and for which questions new clusters should be formulated. Adolescents asked the chatbot an average of 14 questions (including greetings and other comments). The existing chatbot database could be expanded with 14 additional training phrases within existing clusters. Moreover, the chatbot did not give a correct answer to 37 sleep-questions, as determined by the researcher (e.g., a question on sleep received an answer on physical activity, a question on sleep received a response that the chatbot did not understand the question, etc.). These questions were very practical in nature and had not yet appeared from the chat threads and focus groups. However, these questions turned out to be relevant to adolescents (e.g., about sleeping late, dreams, why people have to go to the toilet at night, snoring, taking naps, a morning mood, and so on). These 37 new questions were, on the basis of discussion, divided into several clusters that were added to the existing database. In addition, the pretesting confirmed that adolescents asked many small talk questions (e.g., what day is it tomorrow, what time is it, do you have a sweetheart?). This small talk was not yet extensively included in the initial database.

##### Refine Guiding Principles

Based on the pretesting conversation logs, adjustments were made to the prototype. Two social cues within the verbal category of Feine's taxonomy (38) were expanded, namely increasing small talk to keep adolescents engaged and varying responses to avoid user frustration (as could be deduced from adolescents' reactions: “you don't understand me,” “answer my question,” “never mind,” etc.). Furthermore, new questions that emerged from the conversation logs were added to the database, so that the chatbot could answer more accurately. At the end of this phase, there were ~860 questions processed in the database.

#### Phase 3 With Study 3: Mixed Methods Process Evaluation

During the mixed methods process evaluation stage, the adapted prototype was tested in a real-life setting by the end-users. The modified prototype consisted of the self-regulation app and the chatbot. The video narrative was still under development, so could not yet be included in the pilot study. This study, however, focuses only on the chatbot component. In this stage, qualitative research methods are often triangulated with quantitative methods in order to gain a clear picture of how and why people engage with the intervention (67). Therefore, a pilot study with process evaluation was carried out. The aims of this pilot study were: (1) to assess whether the chatbot worked as expected in a real-life setting (e.g., check whether there were any technical bugs), (2) to gather more input that could be included in the database, and (3) to explore adolescents' objective and subjective engagement with the chatbot. This allows to optimize the chatbot before evaluating its efficacy in future research. The pilot study consisted of three parts. First, the conversation logs were monitored throughout a 2-week intervention period. Second, adolescents filled in a questionnaire exploring their engagement with the chatbot. And third, process evaluation interviews were conducted.

##### Participants

Convenience sampling was used to recruit schools. The schools that already participated in the focus groups in study 1 were excluded in the pilot study to avoid bias. Twenty schools were contacted, three of which agreed to participate in the pilot study (i.e., response rate of 15%). A total of seven classes from these three schools participated. These seven classes comprised a total of 81 adolescents: 43 (53.1%) were in general academic track education and 38 (46.9%) in technical or vocational track education. Adolescents who wanted to participate in the pilot study were given a cinema ticket as incentive. Adolescents who also participated in the subsequent process evaluation interview additionally received a power bank.

##### Procedure

Data collection of the pilot study took place in January 2020. Schools were visited twice. During the first school visit, general information about the project was provided and baseline measures were collected in an online questionnaire. Adolescents were instructed to download the intervention (app and chatbot) on their smartphone. Researchers were present to solve any technical problems during installation. Participants were asked to use the chatbot for 2 weeks. Compared to study 2, adolescents were now informed that they could ask the chatbot questions about the four health domains that the project focused on. After 2 weeks, during a second school visit, adolescents completed post-measures in an online questionnaire, assessing the level of engagement with the chatbot. Subsequently, two students per class were selected by the teacher to participate in a semi-structured interview.

##### Measurements

*Conversation Logs*. Conversation logs between the adolescents and the chatbot were stored that showed the questions the adolescent asked and how the chatbot responded.

*Engagement Questionnaire*. User engagement was assessed at post-usage measurement with items from the Digital Behavior Change Intervention (DBCI) Engagement Scale (items 1, 2, 3, and 6) (22) and the User Engagement Scale (UES) (subscale Perceived Usability, items 1–4) (80). The DBCI is an instrument of 7 items and is assumed to be unifactorial and internally reliable (α = 0.77) (22). A model of 4 items (items, 1, 2, 3, and 6) that consistently showed high loadings on the experiential engagement dimension in the scale development process (22) was chosen, showing good internal consistency (α = 0.85). This was supplemented with the first four items of the subscale Perceived Usability of the UES, as these items pertained to the (negative) emotions experienced by respondents when using the chatbot. The 8 items were rated on a 5-point Likert-scale ranging from 1: totally disagreed to 5: totally agreed.

*Interview Guide Process Evaluation*. The semi-structured interview guide for the process evaluation was based on the Medical Research Council Framework (81) that describes the evaluation of complex interventions. Questions were formulated around three main themes: (1) feasibility, (2) theory of change, and (3) context. To inquire feasibility of the chatbot, adolescents were asked what it was like to use the chatbot (e.g., easy to use, fun to use, accuracy and comprehensibility of the responses, opinion about design, etc.). Theory of change was explored by asking whether the chatbot supported them in any way, and how it affected their behavior. Finally, we examined in which context the adolescents had used the chatbot (e.g., where were you, what were you doing, what made you use the chatbot, etc.). The interview guide can be found in Supplementary Material 7.

##### Analysis

For the quantitative data (e.g., engagement questionnaire), descriptive analyses (means and percentages) were conducted in SPSS 27.0. For the qualitative data (e.g., process evaluation interviews), audio-recordings were transcribed verbatim and transcripts were examined and coded independently by one researcher (LM) and two master's thesis students (ICC = 0.73). All qualitative data were managed in NVivo 12.0.

##### Results

***Conversation Logs** Sample description*. The conversation logs showed that 60 of the 81 participating adolescents tested the chatbot during the pilot phase. Forty participants (i.e., 2/3rd) quit and did not ask any further question after the chatbot had given a wrong answer, as determined by the researcher (e.g., small talk comments got the response that the chatbot did not understand the comments).

*Findings*. In total, 307 questions were asked by 60 adolescents during the 2-week period. An average of 11 questions were asked per participant. There were ~187 new small talk-“questions” (61%). Only 68 (22%) new questions were found in relation to a healthy lifestyle and mental well-being. This shows that especially small talk needs more updating to avoid adolescents dropping out at the start of using the chatbot.

Adolescents also asked 27 (9%) questions related to ambiguities in the app (e.g., how can I set a goal, how can I earn coins, where do I find my sleep results, etc.) and 25 (8%) questions related to other components of the intervention, such as the wearable device (e.g., is the Fitbit waterproof, how do I connect the Fitbit to my mobile phone, how do I synchronize, etc.). Questions relating to technical aspects of the use of the app and Fitbit were not yet included in the chatbot database, because prior to this, the app and chatbot had not yet been tested together in one session.

Adolescents moreover often asked the chatbot whether the chatbot sleeps enough, has breakfast, is physically active, etc. (e.g., do you exercise a lot, do you think exercise is important, how often do you eat breakfast, how much do you sleep, etc.) instead of asking the question about themselves, as if treating the chatbot as a person.

***Engagement Questionnaire** Sample description*. Seventy-three of the 81 participating adolescents completed the engagement questionnaire, of whom 20 had missing descriptive values (e.g., no information on gender, age, educational track). Of the remaining 53 participants, 64.2% were girls, 52.8% were in general academic track education and 47.2% in technical-vocational track education. The mean age was 13.68y (SD = 0.89) and 15.1% was in the 7th grade, 18.9% in the 8th grade, and 66% in the 9th grade.

**Figure 2:**
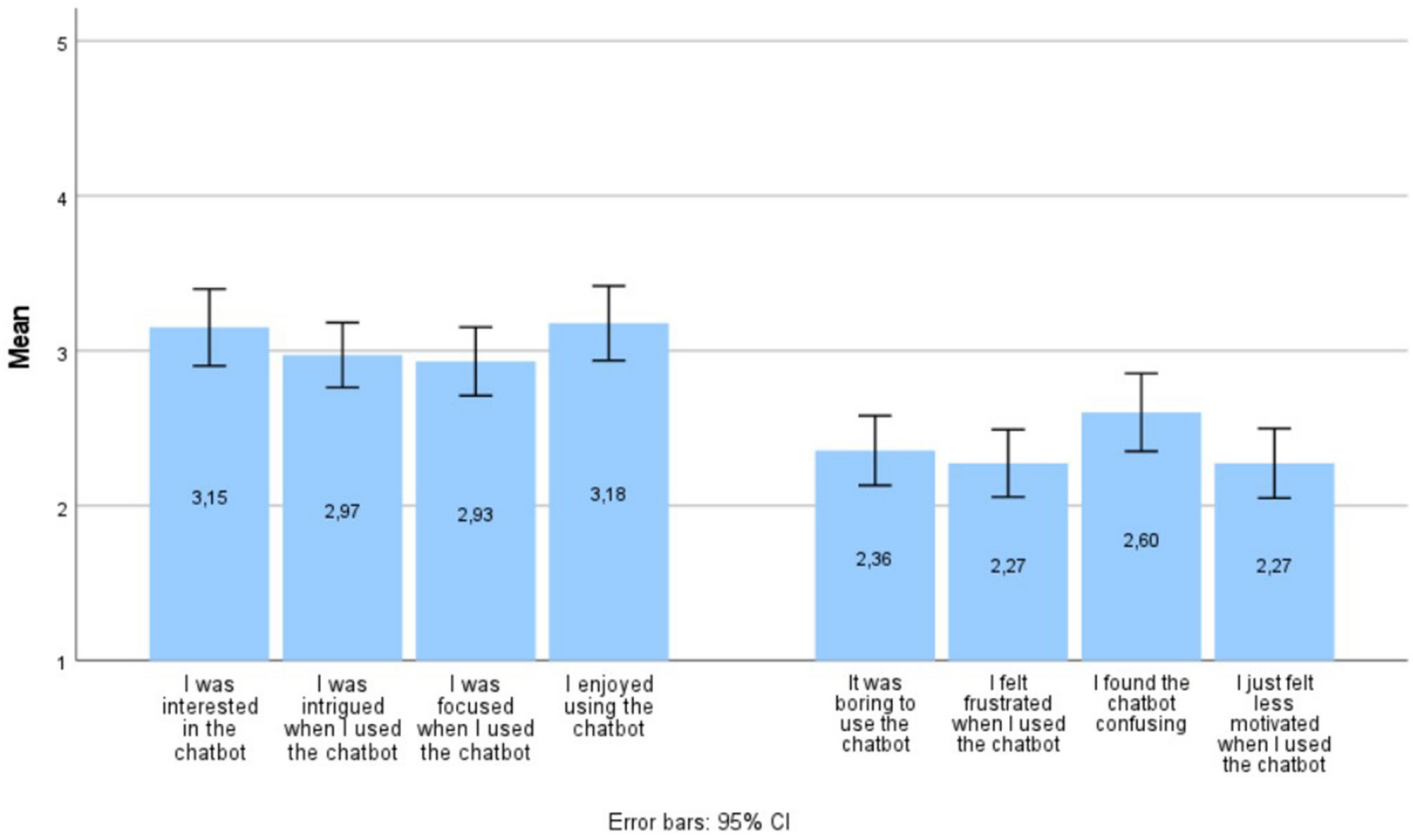
Results (means) on the engagement items during pilot testing.

*Findings*. Results on the engagement items can be found in Figure 2. The positive engagement items (on the left: item 1–4, e.g., I was interested in the chatbot) received neutral scores. Among these positive items, the highest scores were observed for the items indicating that adolescents were interested in the chatbot and enjoyed using it. The negative engagement-items (on the right: items 5–8, e.g., it was boring to use the chatbot) reached an average score between “disagree” and “neutral.” Among the negative items, the highest score was found for the impression that the chatbot was confusing. There was high unanimity among adolescents, as the standard deviations around the averages are quite small.

***Process Evaluation***. Interview duration ranged from 6.5 to 22 min. Duration was limited because adolescents were able to answer the question quickly and did not need much probing and because the time available for the interview was limited as it took place during the remainder of the class hour, after completing the post-test questionnaire.

*Sample description*. A subsample of the pilot study participated in the process evaluation. Thirteen adolescents, among whom 8 girls and 5 boys, were interviewed on the chatbot prototype.

*Findings*. The results of the process evaluation interviews were categorized according to the three main themes of the interview guide: (1) feasibility, (2) theory of change, and (3) context. The main theme “feasibility” was subdivided in (1) content, (2) design, (3) questions, and (4) answers, based on the responses in the focus groups (study 1). Quotes to illustrate the different themes and subthemes can be found in Tables 5, 6.

**Table 5:** Results from the process evaluation interviews on the main theme “feasibility”.

**Theme**	**Subtheme**	**Quote**
**Feasibility—content**	General user experience	“*There were some things that weren't right. But other than that, it was good.” (Girl 3, grade 9, vocational track)*
	Humor	“*His answers were pretty funny” (Girl 1, grade 9, vocational track)*
	English language use	*Interviewer: “what did you dislike about the chatbot?”* *Adolescent: “When you said something, it was like this when you said ‘thank you' [in Dutch]. I thought it was like this, that he started in English.” (Girl 3, grade 9, vocational track)*
	Too redundant responses	“*After my nephew's question of 'I'm sitting too much', the chatbot sent a little too many messages. I would send that in one message. Not so much in different messages. Those notifications came in all the time. So one message that would be enough I think.” (Girl 1, grade 9, vocational track)*
**Feasibility—design**	Nice design	*Interviewer: “if you just look at how the chatbot looked in your app. What did you think of the view?”* *Adolescent: “I liked that. Also nice that you can change the background.” (Girl 7, grade 7, vocational track)*
	Settings page was not visible enough	“*Nah I didn't do that. I see this just now…whoops” (Boy 2, third year, general academic track)*
**Feasibility—questions**	Small talk	“*I tested the chatbot, I asked if it could calculate something, but it didn't work.” (Girl 8, grade 7, general academic track)*
**Feasibility—answers**	Answers were not always accurate	“*Erm, I used it once. The first day I asked if I had to keep the Fitbit on at night because I didn't know. Erm, it was a bit weird but erm, mainly because I don't think the answers were on point yet because I got some really weird answers. I got links to videos that helped me fall asleep better.” (Girl 4, grade 9, vocational track)*
	Drop-out in case of wrong or strange answers	“*Um, I've only used the chatbot twice or so. I had asked something about 'how could you earn coins' [i.e., part of the app] and it gave an answer that was not clear. And then I thought well, I don't need to use it.” (Girl 5, grade 9, vocational track)*
	Immediate response	“*And that he answers so quickly. You have sent a message and 2 seconds later you have your answer back already. That's nice, that you don't have to wait that long.” (Girl 1, grade 9, vocational track)*
	Referral to website	“*I thought it was good. I used the little robot. I wrote something down and then suddenly I was sent to a website. There I could see what I wanted to know.” (Boy 3, grade 9, vocational track)*
	Length of responses	“*Rather short answers than long texts. Two lines is enough” (Boy 2, grade 9, general academic track)*

**Table 6:** Results from the process evaluation interviews on the main themes “theory of change” and “context.”

**Theme**	**Subtheme**	**Quote**
**Theory of change**	No support	*Interviewer: “Did the chatbot help you with anything?”* *Adolescent: “No, it didn't.” (Boy 1, grade 9, vocational track)*
	Social support	“*Um, that he can encourage you more to do something” (Girl 2, grade 9, general academic track)*
	Instrumental support	“*So I asked uhm, yes how did I ask it, ‘can you help me move more' and then he answered with a site and a text with more information… yes you can find all kinds of things because of the chatbot.” (Boy 2, grade 9, general academic track)*
	Emotional support	“*Um (…) the chatbot helped me the most because I was in a whole emo-dip last week and I had asked 'what should I do with that?' Then he answered 'maybe you should watch some happy videos' and I thought okay, maybe I should do that sometime. I tried that and it helped sort of, a little bit.” (Girl 1, grade 9, vocational track)*
	Support with the app	“*If there are uncertainties about, for example, the secret agents [i.e., part of the app], you might really ask the chatbot 'what is the intention?' So maybe that's very useful.” (Girl 4, grade 9, vocational track)*
**Context**	After school	“*I used the chatbot after school, at 4-5pm.”* *(Boy 5, grade 8, general academic track)*

Within the theme “feasibility” (Table 5), adolescents generally reported having a good experience with the chatbot. They recognized and appreciated the humor in the chatbot's replies. The design was appreciated as well. However, there was also room for improvement. For instance, some chatbot answers were inadvertently provided in English (while the chatbot was programmed in Dutch). Moreover, longer answers were considered annoying by adolescents, because this meant having to scroll down to read all the information. Furthermore, the settings page was not sufficiently clear for the users. Adolescents reported they first tested the chatbot using small talk. They further noted that answers were not always accurate (i.e., mismatch between question and response), and they subsequently stopped using the chatbot. Users also appreciated receiving immediate responses to their questions and being directed toward additional information on websites. Opinions differed on the degree of support the chatbot could offer them. Some experienced no support, others experienced social, instrumental, emotional and/or support with the app. Table 6 shows examples of the various forms of support. Within the theme of “context,” it was clear that adolescents mainly used the chatbot right after school.

## Discussion

The aim of this study was to describe the research findings that guided the development of a chatbot for youth mental health promotion using the Person-Based Approach. This approach gains an in-depth understanding of adolescents' perceptions prior to and throughout intervention development, in consecutive phases, to create a more engaging intervention (42–44). Several key findings emerged from this PBA study: (1) a number of adolescent preferences were identified that were not previously described in literature, (2) this systematic process (e.g., preference elicitation prior to development, reflections on prototype in qualitative research, testing of prototype in real-life setting) provided new insights for youth chatbot preferences and (3) despite this meticulous approach and our efforts, the user engagement with the chatbot was still moderate. In what follows, we will discuss these three key findings in more detail.

The first key finding involves the emergence of a number of preferences that, to the best of our knowledge, have not been previously described in adolescent chatbot literature. User preferences showed youngsters are concerned about confidentiality: they wanted a trustworthy chatbot in which they can delete the conversation so they can protect their privacy. This concern about Internet safety among youth that has not appeared in chatbot literature for a general (adult) population, may be understood from the attention paid in many school curricula to safe Internet practices. These have mainly appeared in school curricula around 2,000 (82, 83), so most adults may not have benefited from such courses when they were at school. Consequently, it could be the case that youth have grown up more with netiquette and potential dangers of digital media than adults (84), resulting in being more cautious (85). Furthermore, adolescents wanted a chatbot that fits with their personal life and youth culture: one that does not treat them in a childish manner, uses youth language (e.g., emojis), has a design similar to message apps they know and often use, allows them to ask questions about what is relevant to them (also non-health related), and formulates realistic answers in a positive way. This suggests that even if certain preferences for features are shared between youth and general populations, chatbots for adults may not be a perfect fit for youth. Youth chatbots should be designed taking youth-cultural specificities into account. Youngsters, however, also share a number of preferences with what is known from literature in a general population: adolescents prefer a chatbot they can ask unlimited questions to (i.e., free dialogue); a chatbot that allows small talk and fast responses; an empathetic, humoristic, and anonymous tool with a personality to which they can address questions they find difficult to ask others. Other design preferences for the chatbot, such as a design with a cheerful (e.g., colorful) form and the ability to personalize (e.g., changing backgrounds) are consistent with what is known from youth preferences for other digital interventions such as games and apps (86).

The focus of this chatbot was on primary prevention via the promotion of healthy lifestyles of physical activity, low sedentary behavior, sleep and diet, rather than treatment of mental health problems. This was addressed by referring to sources where adolescents could find appropriate information or help. Adolescent opinions were mixed regarding referring to other sources or websites. Some indicated that this was not a real answer to their question, and that they would prefer the chatbot to answer directly. Others mentioned that this referral increased the reliability of the chatbot replies. It may be advisable to make the website links for further info or care optional, and to have at least a basic response within the chatbot. Moreover, adolescents indicated a preference for a direct link to a webpage with the relevant information rather than to a general website.

After conducting the three studies, the taxonomy of social cues for conversational agents (38) enables to identify three categories and five subcategories of social cues that our chatbot may exhibit. There were in total 25 social cues that could be implemented in the design of the chatbot. See Supplementary Material 8 for an overview.

Our second key finding is that this systematic development process (i.e., PBA) involving adolescents in different phases has led to insights that may not have emerged if adolescents had only been involved in the initial phase that broadly identified their preferences (i.e., focus groups in phase 1). Perhaps the most important finding is that through all stages (i.e., the focus groups, log data analysis and pilot study) many adolescents first used or tested the chatbot by making small talk comments. During the focus groups, this only appeared to a small extent. When exploring what questions they would ask the chatbot, adolescents' input was rather limited. When, in later phases (i.e., phase 2 and 3) adolescents could actually test the chatbot on their own smartphone, without the presence of the researchers, the use of small talk became much more prominent. Similar results were found in a chatbot study by Crutzen et al. (51) which focused on answering adolescents' questions about sex, drugs and alcohol. During these conversations, four times as many queries were about other topics than sex, drugs or alcohol (e.g., exchanging greetings). Knowing that adolescents first expect small talk when interacting with a health chatbot has important implications. If the chatbot is not able to make small talk, adolescents may drop out due to frustration before they even get to the core purpose of the chatbot, which is to answer health-related questions (44, 87). Two directions could be taken from here: (1) clarify the purpose of the chatbot to adolescents from the outset, for example by stating which topics it covers when greeting the user to avoid the expectation of small talk (35, 44, 46, 87) or (2) providing the opportunity for small talk in the chatbot. Our findings indicate that the second option may be more fruitful with youth. First, adolescents in our study expected small talk, even if it was made clear at the start that the chatbot handled topics on healthy lifestyles. Moreover, studies showed that users interact with conversational agents in the same way as they would with humans, as also indicated in the CASA paradigm (38, 77, 78). This emphasizes that a more human-like interaction (i.e., the use of social cues) would be more engaging to users. Indeed, when users see conversational agents as companions, they are more inclined to continue interacting with these chatbots (48, 50). A possible explanation for the high importance of the social cue “small talk” in youth is that the current generation of adolescents has grown up with technology, so their expectations of its possibilities may be higher than those of adults. In line with this hypothesis, it could also be that adolescents more often focus on testing the limits of the offered technology, whereas adults perhaps immediately focus more on the purpose of the tool (88). Based on these considerations, it may be advisable to develop the chatbot in such a way that it can optimally respond to the small talk comments adolescents use.

Another finding that only emerged by testing the chatbot in multiple phases, and especially so during testing in a real-life usage context, is that adolescents also had questions for the chatbot about how to use the app and the associated Fitbit. This illustrates that participants also used the chatbot to receive assistance with and information about the broader intervention (87). As a result, in addition to the social support function for which it was intended, the chatbot could have a more instrumental function in which it could assist adolescents with ambiguities and difficulties in the app and with the Fitbit.

Furthermore, the prototype testing in real-life provided the additional benefit of not eliciting adolescent preferences in a hypothetical, but in a real, concrete situation. Several suggestions on possible questions adolescents would ask were collected during this real-life prototype testing that had not come up during the focus group sessions. Focus group sessions may have created barriers due to social desirability, or may have been too abstract, despite the use of prompting material (i.e., example questions and answers from the chat threads, visual examples, additional explanations when the question was not understood). Especially for adolescents in technical-vocational education, focus group discussions on hypothetical situations seemed to be difficult. Conducting formative research and user-testing of digital interventions in different cycles including real-life situations is therefore warranted. This can reveal insights that would not come to light in a one-stage testing.

Our third key finding showed that, despite our extensive PBA development process, user engagement with the chatbot still appeared to be only moderate. The scores on subjective engagement with the developed chatbot were not overwhelmingly positive, and the log data analysis and process evaluation interviews revealed that when the chatbot did not meet adolescents' expectations, many adolescents stopped using the chatbot due to frustration. The engagement questionnaire moreover showed that the chatbot was experienced as confusing to some adolescents, possibly due to the mismatch between the user's question and the chatbot response. This mismatch is a limitation from the choice to use a Natural Language Processor (NLP), in comparison to constrained input used in the majority of interventions where the input is rule-based, giving people different answer-options to choose from in order to shape the conversation (29, 34). The NLP's task is to extract the semantic representation of users' comments, so that corresponding responses can be returned. NLP is a critical component and one of the biggest challenges in chatbot development (57). Previous studies showed that failures in the NLP were the greatest factor in users' negative experience with conversational agents, leading to user dissatisfaction in a quarter of cases (89). The choice to use NLP was made as adolescents preferred free language input, but it presented several challenges. First, although the researchers integrated as many adolescent comments as possible into the software, it was impossible to anticipate and prepare the chatbot for every possible small talk comment the test users could make. Second, adolescents made spelling, grammar or typing mistakes or used synonyms and youth language in their question. For example, the shortening of words: “hayd?” instead of “how are you doing?” or a question about a specific sport (i.e., golf), whereas only other sports were included in the input, resulted in a failure by NLP to recognize the question and resulted in an incorrect response. This phenomenon has also been described in previous studies (29, 44, 57, 87). Specifically for adolescents, prior work demonstrated that adolescents found it difficult to phrase their questions in such a way that the chatbot understood them (51). Natural language is moreover very context-dependent; the same comment may have a very different meaning in a different context (30, 57). For example, if while greeting the chatbot adolescents reply with “bad” to the question of how they are doing, the chatbot cannot deduce in what context adolescents have now replied with “bad.” They might just as well answer “bad” when asked how they sleep. Although technology is a rapidly changing field, and advances are made, future research needs to focus on mismatches between adolescent comments and the (extensiveness of the) chatbot database. This could be done by a constant updating of the database based on real-time use (“living database”).

Based on our key findings, we can conclude that this extensive participatory development process has certainly led to new insights, but this process cannot be called superior since the outcome (i.e., engagement) did not show better results. A participatory development process is important, but this approach might be supplemented with strengths from other approaches in the future. For example, the participatory approach can be complemented by a crowdsourcing-based approach where information can be collected from a larger group of users via microtasks (90–92). A limitation of the PBA seems to be that only a limited number of users can be surveyed in depth. Consequently, the use of convenience sampling strategies result in not being able to map out all the preferences from the target group, providing only a very limited picture of the reality. Counterbalancing this with a crowdsourcing-based approach, where more users can be reached, could be an added value to tailor the chatbot as much as possible to the entire target audience. In addition, this could also be complemented by a data-driven bottom-up approach where existing data can be analyzed in order to be able to make the chatbot more intelligent, for example through developing personas using algorithms so that the chatbot can answer in a much more tailored way (93, 94).

While NLP appeared as a good yet technically challenging choice for our (mental) health promotion chatbot, providing a wrong answer when confronted with mental health disorders presents an important risk of creating harm (34, 35, 56, 57). Although treatment and support with mental health disorders was not the focus of our chatbot, additional precautions need to be taken if applying our approach to mental health treatment.

### Limitations and Strengths

This study has a number of limitations. First, the study is limited by its sampling method (i.e., convenience sampling) and by having been conducted only in Flanders, which reduces the generalizability of our results to other countries and settings. Mainly girls were represented in our samples and most input was provided by adolescents from the general academic track. Efforts were made to reach diversity in the socio-economic background of our sample. The Flemish Health Behavior in School-aged Children (HBSC) study (95) has demonstrated that children from lower family affluence are more often represented in vocational and technical schools (i.e., non-academic educational tracks), whereas children from higher family affluence are more often represented in academic track education. Therefore, in each of the three studies, an attempt was made to recruit schools from both the academic and non-academic educational tracks, aiming to include adolescents from different socio-economic family backgrounds. In study 1 and study 3 there was an approximately equal mix of both educational tracks. In study 2, the educational track of the participating adolescents was unknown. Although efforts were made to involve adolescents of a non-academic educational track, their proactive input was rather limited. This was partly overcome by testing the chatbot in a real-life setting during the third study, which reduced problems of literacy and need for abstract thinking as in focus groups. However, due to their relatively limited input, we cannot state with certainty that the developed chatbot will sufficiently match the preferences and needs of adolescents from non-academic educational tracks. Second, an inclusion criterion was a sufficient knowledge of Dutch, to be able to use the chatbot and fill out surveys in Dutch. In the region where this study took place, all education is provided in Dutch and none of the potential participants were excluded due to a lack of knowledge of Dutch. However, we did not include schools that provide education for recent immigrants, who are just starting to learn the basics of the Dutch language. Our study results may therefore not generalize to this specific population of recent immigrants who do not yet master Dutch. Third, some adolescents who completed the post-test questionnaire had not used the chatbot (*n* = 13), and the log data did not allow to identify these individuals, resulting in a biased opinion for this small group. Fourth, log data was monitored in this study, but not in much detail. Future research could focus even more closely on the log data, for example by examining how many questions the chatbot answered accurately, how well the chatbot was used as compared to the rest of the app, etc. The main strength of this study is that the input for the database was developed in a fully participatory manner in accordance with a theoretical framework, the PBA. This systematic approach involved adolescents at different stages of development and allowed them to work with real material and not just abstract ideas. Throughout the entire development of this chatbot, the starting point was the adolescent him- or her-self.

## Conclusion

This paper described the extensive development process of a health promotion chatbot for youth, using the PBA. New results that had not been described in previous studies included the importance of confidentiality, connection to youth culture, and preferences when referring to other sources. Developing a chatbot is an iterative process, in which repetitive testing with the target group is required. The systematic development process allowed for additional insights to emerge such as the importance of small talk for this user group, the wider support (e.g., technical issues) the chatbot could provide than just social support regarding healthy lifestyle behaviors and the combination of focus groups with real-life testing also proved useful to include the perspective of youth from non-academic educational tracks. Engagement with the chatbot turned out to be modest. Using living databases is needed to counteract the challenges of NLP, to advance the quality of chatbots for youth health promotion.

## Data Availability Statement

The raw data supporting the conclusions of this article will be made available by the authors, without undue reservation.

## Ethics Statement

The studies involving human participants were reviewed and approved by the Committee of Medical Ethics of the Ghent University Hospital (Belgian registration number: EC/2019/0245) and the Ethics Committee of the Faculty of Psychology and Educational Sciences of Ghent University (registration number: 2019/93). Written informed consent to participate in this study was provided by the participants' legal guardian/next of kin.

## Author Contributions

LM, AD, and CP: conceptualized the study. LM and CP: collected the data. LM: wrote the original draft. AD: double-coded the data. AD, CP, GCa, GCr, and SC: edited the manuscript and provided feedback. All authors read and approved the final manuscript.

## Funding

This work was supported by the Flemish Agency for Care and Health. LM was funded by Research Foundation Flanders (Grant Number: 11F3621N, 2020–2024). This funding body is, however, not involved in the study design, collection, management, analysis, and interpretation of data or in writing of the report.

## Conflict of Interest

The authors declare that the research was conducted in the absence of any commercial or financial relationships that could be construed as a potential conflict of interest.

## Publisher's Note

All claims expressed in this article are solely those of the authors and do not necessarily represent those of their affiliated organizations, or those of the publisher, the editors and the reviewers. Any product that may be evaluated in this article, or claim that may be made by its manufacturer, is not guaranteed or endorsed by the publisher.

## Footnotes

^1^https://www.pandorabots.com/mitsuku/.

^2^https://dialogflow.com/.
