# Supplementary material for: Participatory Development and Pilot Testing of an Adolescent Health Promotion Chatbot
Source: Front Public Health. 2021 Nov 11;9:724779. doi: 10.3389/fpubh.2021.724779 (PMC8632020; doi:10.3389/fpubh.2021.724779)
Supplement: Supplementary file 1 [file Data_Sheet_1.DOCX]

Supplementary material

Content

[Supplementary file 1 2](#_Toc81484519)

[Screenshots of the #LIFEGOALS app 2](#_Toc81484520)

[Supplementary file 2 3](#_Toc81484521)

[Example of social cues for conversational agents, based on taxonomy of Feine et al.[3] 3](#_Toc81484522)

[Supplementary file 3 4](#_Toc81484523)

[Analysing chat threads of a Flemish online helpline for youth 4](#_Toc81484524)

[Supplementary file 4 6](#_Toc81484525)

[Interview guide focus groups 6](#_Toc81484526)

[Supplementary file 5 10](#_Toc81484527)

[Social cues that emerged from the focus groups and that were added to the cues found in literature 10](#_Toc81484528)

[Supplementary file 6 11](#_Toc81484529)

[Screenshots from the chatbot prototype 11](#_Toc81484530)

[Supplementary file 7 12](#_Toc81484531)

[Interview guide process evaluation 12](#_Toc81484532)

[Supplementary file 8 13](#_Toc81484533)

[Overview of the social cues embedded in the chatbot 13](#_Toc81484534)

# Supplementary file 1

## Screenshots of the #LIFEGOALS app

*
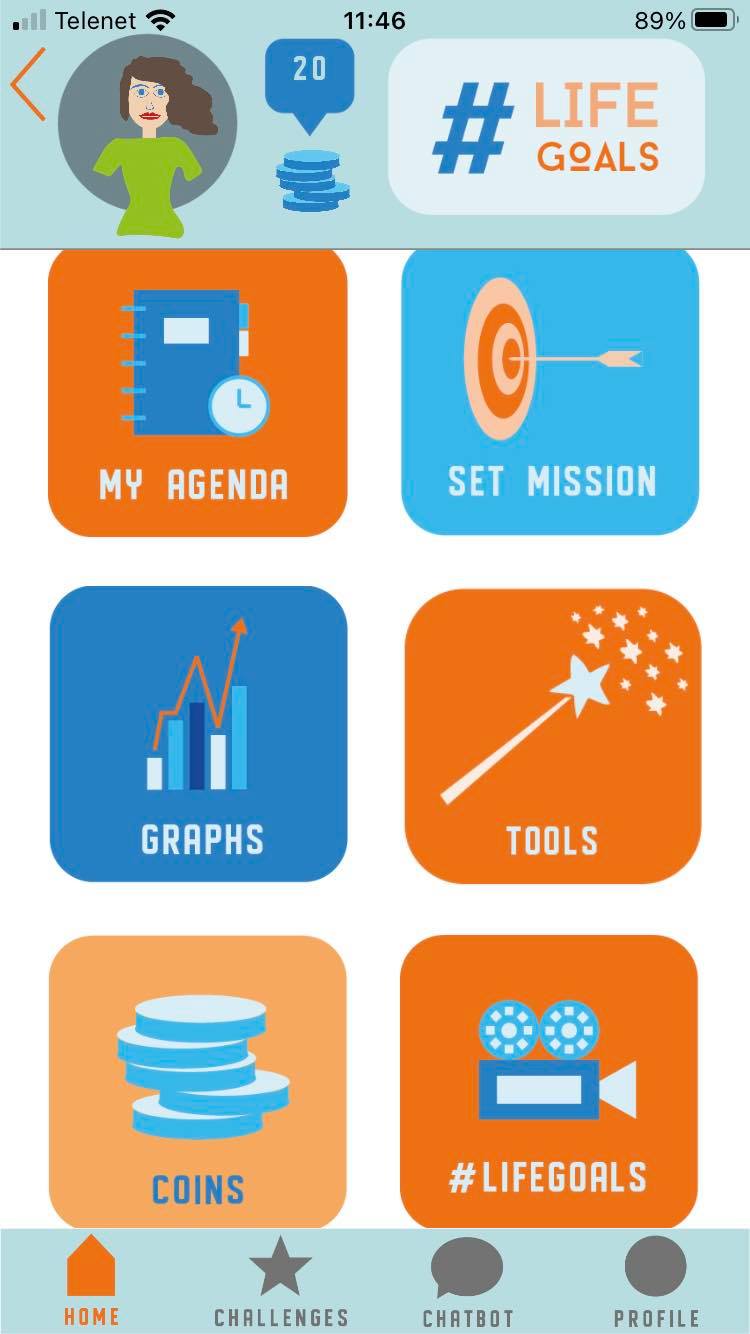

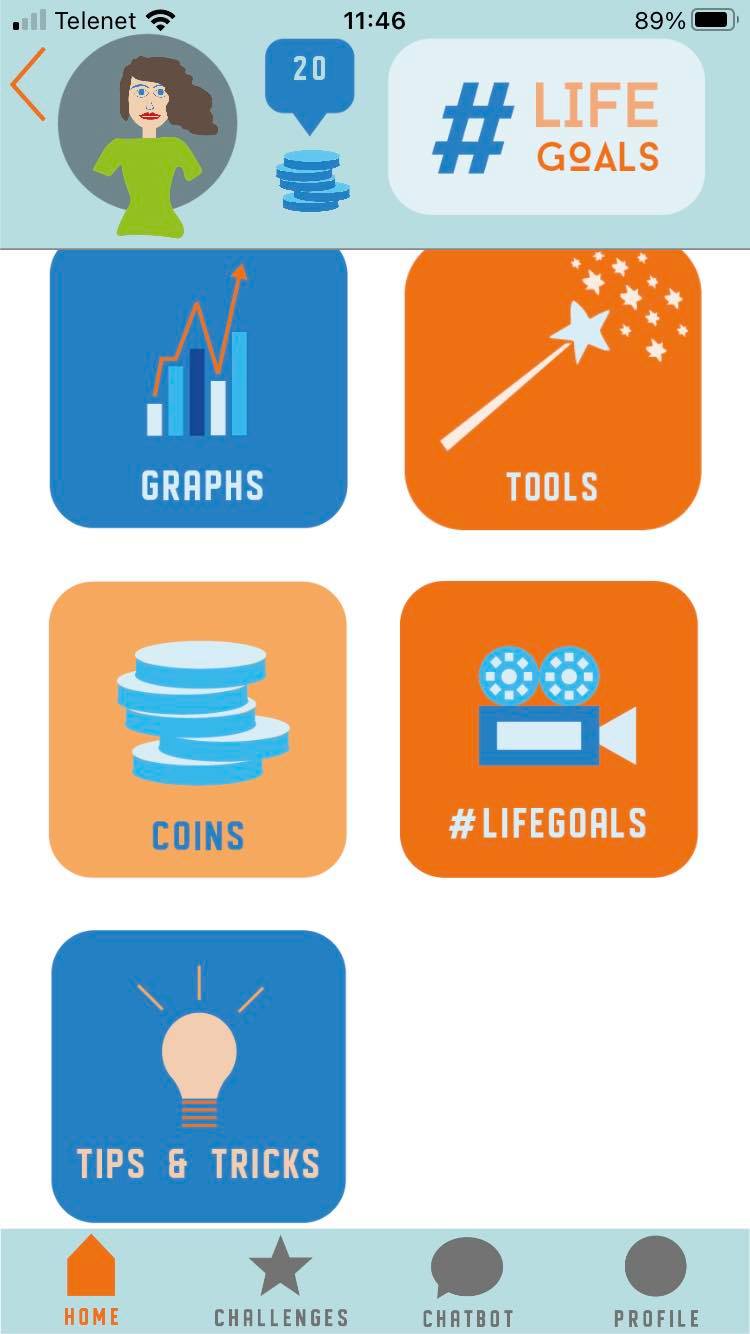
*

# Supplementary file 2

## Example of social cues for conversational agents, based on taxonomy of Feine et al.[3]

| **Category** | **Subcategory** | **Social cue** |
| --- | --- | --- |
| 1. **Verbal** | *Content* | Use of humor and empathy, engage in small talk, appropriate referrals in case of safety-critical health issues, express a name, overall chatbot description, asking questions related to topics that are difficult to talk about with their parents |
|  | *Style* | Short and precise interaction, variation in system responses and dialogue structures, anonymity, free dialogue, avoid redundant answers |
| 1. **Visual** | *Kinesics* |  |
|  | *Proxemics* |  |
|  | *Agent appearance* | Profile picture, personality |
|  | *Computer-mediated communication (CMC)* |  |
| 1. **Auditory** | *Voice qualities* |  |
|  | *Vocalizations* |  |
| 1. **Invisible** | *Chronemics* | Fast responses |
|  | *Haptics* |  |

# Supplementary file 3

## Analysing chat threads of a Flemish online helpline for youth

*Procedure*

To enrich the content of the message database underlying the current health promotion chatbot, anonymised real life chat- and email conversations on health behaviours linked to mental well-being between adolescents and volunteers of an online helpline for youth (i.e., *Awel*) were read. By doing so, insight was obtained into 1) the questions adolescents asked regarding the included health behaviours (e.g., physical activity, sedentary behaviour, sleep and healthy diet), 2) what language (e.g., word choice, synonyms, abbreviations, etc.) they used to ask their questions, 3) the answers/suggestions the volunteers gave or what referrals they were making, and 4) whether adolescents felt that this helped them in any way.

The content for this chatbot was selected based on a number of themes of the ‘*Awel’*-database itself. The themes that were selected were relevant for the intended purpose of the chatbot (i.e. health behaviours linked to mental well-being), namely: physical health and appearance, mental health, hobbies, boredom, identity, school results, exam stress/work pressure/fear of failure, motivation/school fatigue, and social media.

Chats and email conversations from the period January to September 2018 were read using NVivo to categorize them in the different health domains. Based on all selected themes from the ‘*Awel’*-database, 2035 chat threads and emails were retrieved at first glance (see figure 1). In a next step, we drew up key words for each health domain (e.g., for physical activity: move(ment), sport(s), activity, active, etc.) with which we went through these conversations. If a key word occurred, the conversation was retained. If the keyword did not occur, the conversation was removed. This led to 469 remaining conversations. Subsequently, all conversations were briefly read through. If the conversation contained information about one of the four included healthy lifestyle behaviours (e.g., physical activity, sedentary behaviour, sleep and healthy diet), and a link could be made with adolescents’ mental well-being, the conversation was retained. This led to 319 remaining conversations that were reread and analysed using NVivo (see Figure 1).

Figure 1

These conversations served mainly to shape the database of the chatbot (e.g., how do adolescents ask questions about a healthy lifestyle? What word choice, synonyms, abbreviations, sentence structure, etc. do they use?) and to draw up the interview guide for the focus groups.

# Supplementary file 4

## Interview guide focus groups

**Welcome & introduction [2 min.]**

Hi all, thanks for joining this group discussion. I am ... and this is ..., and we are researchers at Ghent University in the department of Movement and Sports Sciences.

Before we start, I would like to go over some guidelines on how this focus group works.

- We are purely interested in your opinion about a chatbot (more on that later). So there are no right or wrong answers, just different opinions, and for us every opinion is important and okay.
- You may have noticed the little recording device. We record the conversation so that we don't forget anything afterwards. Very interesting things are often said in these discussions and we cannot write fast enough to put it all on paper.
- I would ask that no more than one person speaks at a time, otherwise we will not be able to understand each other properly. You don't have to agree with others, but you do have to listen with respect when others share their views.
- If that is not the case yet, I would like to ask you all to put your mobile phones on silent so that we are not distracted in between.
- There are name tags in front of you to make it easier for me to direct the conversation, but we promise not to use your names in reports or otherwise.
- My role is that of a moderator, which means that I will try to let all those who have something to say speak. However, you should not try to make something clear to me, but rather to talk to each other.

Are there any questions yet?

1. [8 min.] We are currently working on a project called MOV-E-STAR. With this project, we want to make adolescents feel better about themselves, both physically and mentally, by supporting them in things they can do themselves every day, such as exercising enough, not sitting too much, sleeping enough and eating breakfast every day.

One of the things we are going to do in this project is develop a chatbot. Does anyone know what that is? *[wait for response]*

It's a system on for example a mobile phone that can send you messages and who you can ask things to, and who then gives an automatic answer. Because this chatbot will be used by adolescents like you, between 12 and 15 years old, we would like to ask your opinion about what the chatbot should send, in what words, and how it should be able to respond to your questions.

Example MITSUKU https://www.pandorabots.com/mitsuku/. What could we ask the chatbot about one of these four healthy lifestyles? Then we can see what it answers.

[TESTING]

1. Have you ever used such a chatbot for anything, not necessarily health? What were your experiences, do you still use it, why?

We would also develop something like that within this project.

- 1. Would you actually use such a chatbot?
  2. What do you think this chatbot should be able to do? What would you like?
  3. And what should it absolutely not do? What would you find weird or unpleasant?

1. [5 min.] How would you like this chabot to communicate with you?
   1. What kind of language should the chatbot use?
      1. (Should it use your kind of SMS (youth) language (e.g. dntknw), or better in full sentences like teachers write (e.g. I don't know)?
      2. Emoji's?
   2. How personal should the chatbot be?
      1. (Would you like to feel like it knows you, and maybe remembers some of your previous questions, or does that not matter?)
   3. A friend can show sympathy if you are feeling sad, for example. How much sympathy should or should the chatbot not show?
2. [7 min.] EXAMPLES QUESTIONS ADOLESCENTS, based on chat threads (Each question printed on A4 - put in circle with adolescents)

We have some examples of what adolescents might ask the chatbot.

- 1. "I don't eat breakfast, is that a bad thing?"
  2. "I actually want to lose weight. I weigh quite a lot but I never really have motivation to go for a walk or do exercises or not eat that chocolate sandwich. Do you have any tips?"
  3. "I am often insecure about myself and after eating I sometimes dare to vomit".
  4. "I often lie awake at night worrying about things and therefore I don't sleep well anymore. What can I do?"
  5. Would you please tell me why I have become so lazy and how I can avoid it?
  6. "I very often want to exercise as much as possible but I don't have so much energy anymore."
  7. "I would like to talk about my problems.

Are these questions that you would ask the chatbot?

If you were to ask such a question, what would you expect to receive in response?

- 1. What would you not like at all?
  2. What would still be okay?
  3. What is ideal?

1. [5 min.] What questions would you ask the chatbot?
   1. If you were to ask such a question, what would you expect in terms of an answer?
2. [10 min.] EXAMPLES CHATBOT ANSWERS, based on chat threads (Each answer printed on A4 - put in circle with adolescents)
   1. "Not eating breakfast is not good for your body. You don't get enough vitamins, which can make you feel tired or make you feel less good about yourself! After all, your body needs those nutrients, otherwise it cannot function."
      1. Assume the chatbot says that sentence: what do you think?
      2. Is such an answer OK? What is not OK? What is the best?
   2. "Breakfast is an important meal because you need the energy to start your day."
      1. This is a shorter answer than the previous one. What do you prefer?
   3. "On this website you can also find a lot of tips on sleeping: https://www.noknok.be/vragen-over/slaapproblemen and this one: https://jongerengids.be/vraag/ik-val-moeilijk-slaap-wat-kan-ik-doen"
      1. What would you think if you were sent a link to a website with more information?
      2. Is such a response okay? What is not okay? What is the best?
      3. Would you look at it immediately, or afterwards?
   4. "It is important to reflect on what you think about when you cannot sleep. It is best to discuss these things with a good friend, your parents, or someone else you trust. The longer you sit alone with these things, the longer they will remain in your head. Talking can be a real relief!”
      1. What do you think about the chatbot indicating that you should talk about what's on your mind?
      2. Is such a response okay? What is not okay? What is best?
   5. “What do you think about consulting JAC *(i.e., youth advice centre in Flanders)*? I will send you a link. The JAC is a place where you can mail, chat, call and you can also walk in for a chat. They will then send you on or start a short term counselling session with you: https://www.caw.be/jac/contacteer-ons/"
      1. What would you think if the chatbot would say something like that, that you would be referred to e.g. the JAC, CLB, Awel, a family doctor or a dietician?
      2. Is such a response OK? What is not OK? What is best?
   6. "You may be able to take your mind off things by doing other things, such as walking, playing sports, ..."
      1. What do you think about the chatbot saying something like this?
      2. Is such a response okay? What is not okay?
3. [3 min.] What if you ask the chatbot a question, but it can't give you an answer (because it's not programmed), how do you expect the chatbot to respond?
   1. For example, *'sorry, I don't understand your question. What exactly do you mean?*'
4. [3 min.] The project is about healthy living. How would you feel if the chabot gave you some encouragement? For example, *'just keep going, you are doing well'*?
   1. What kind of encouragement would motivate you to live healthier?
5. [2 min.] What should the chatbot look like?
   1. What should the chatbot be called? Should it have the name of a real person?
   2. What do you think about the name/looks of the chatbot being adapted to the characters of the narrative we are going to make?
6. [2 min.] The aim of this conversation was to gain insight into what you think are good answers from a chatbot in response to questions from adolescents, and which questions the chatbot should definitely be able to answer. Is there anything else that has not been covered?

**Closure [1 min.]**

Then it only remains for us to thank you for your efforts and for sharing your opinions. This helps us a lot in our research and to develop the chatbot. If you are interested in seeing the results of the research, you can send us your email address. *[A paper is passed around on which students can write down their email address without any obligation].*

# Supplementary file 5

## Social cues that emerged from the focus groups and that were added to the cues found in literature

| **Category** | **Subcategory** | **Social cue** |
| --- | --- | --- |
| 1. **Verbal** | *Content* | Use of humor and empathy, engage in small talk, appropriate referrals in case of safety-critical health issues, express a name, **clear overall chatbot description**, asking questions related to topics that are difficult to talk about with their parents, **non-judgmental, trustworthy, questions broader than the chatbot’s purpose** |
|  | *Style* | Short and precise interaction, variation in system responses and dialogue structures, anonymity, free dialogue, avoid redundant answers, **ability to follow the conversation, memorize previous conversations, youth language, notifications, accurate and realistic answers formulated in a positive manner** |
| 1. **Visual** | *Kinesics* |  |
|  | *Proxemics* |  |
|  | *Agent appearance* | Profile picture, personality, cheerful with the ability to personalize |
|  | *Computer-mediated communication (CMC)* | **Emoticons** |
| 1. **Auditory** | *Voice qualities* |  |
|  | *Vocalizations* |  |
| 1. **Invisible** | *Chronemics* | Fast responses |
|  | *Haptics* |  |

# Supplementary file 6

## Screenshots from the chatbot prototype


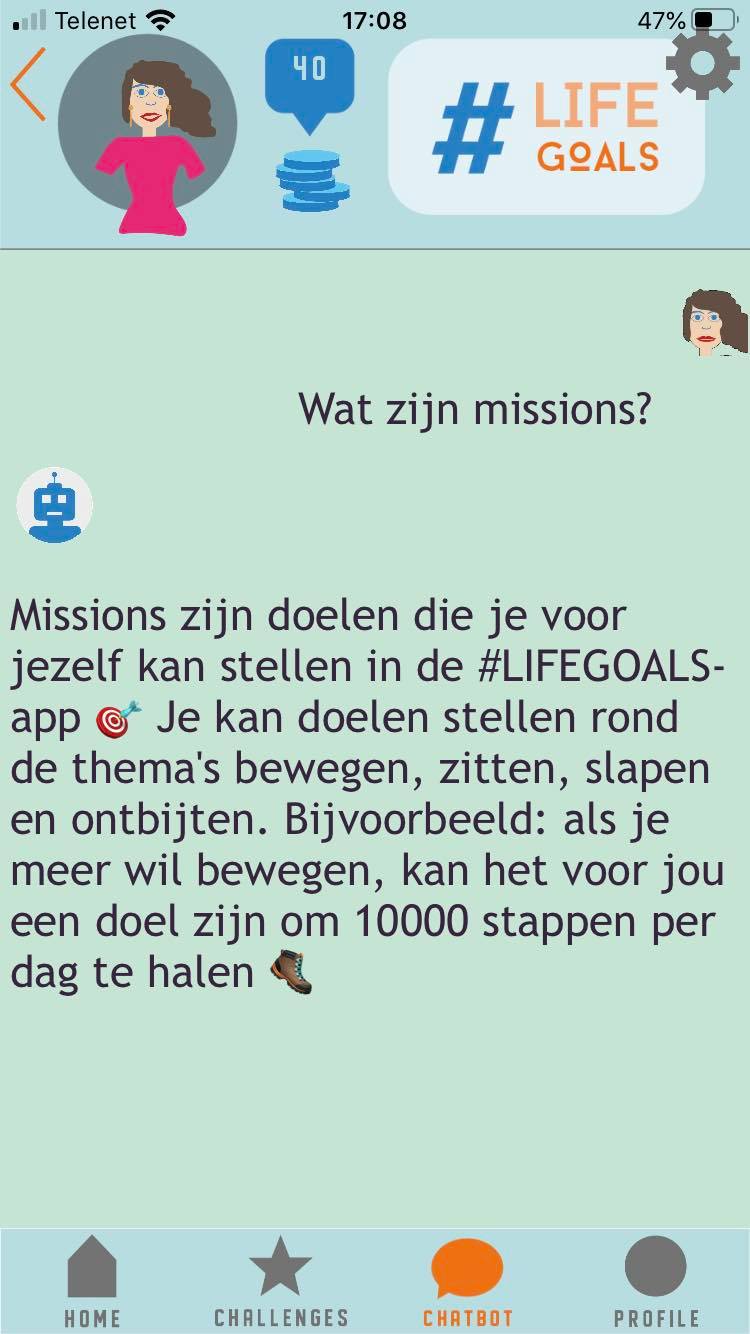


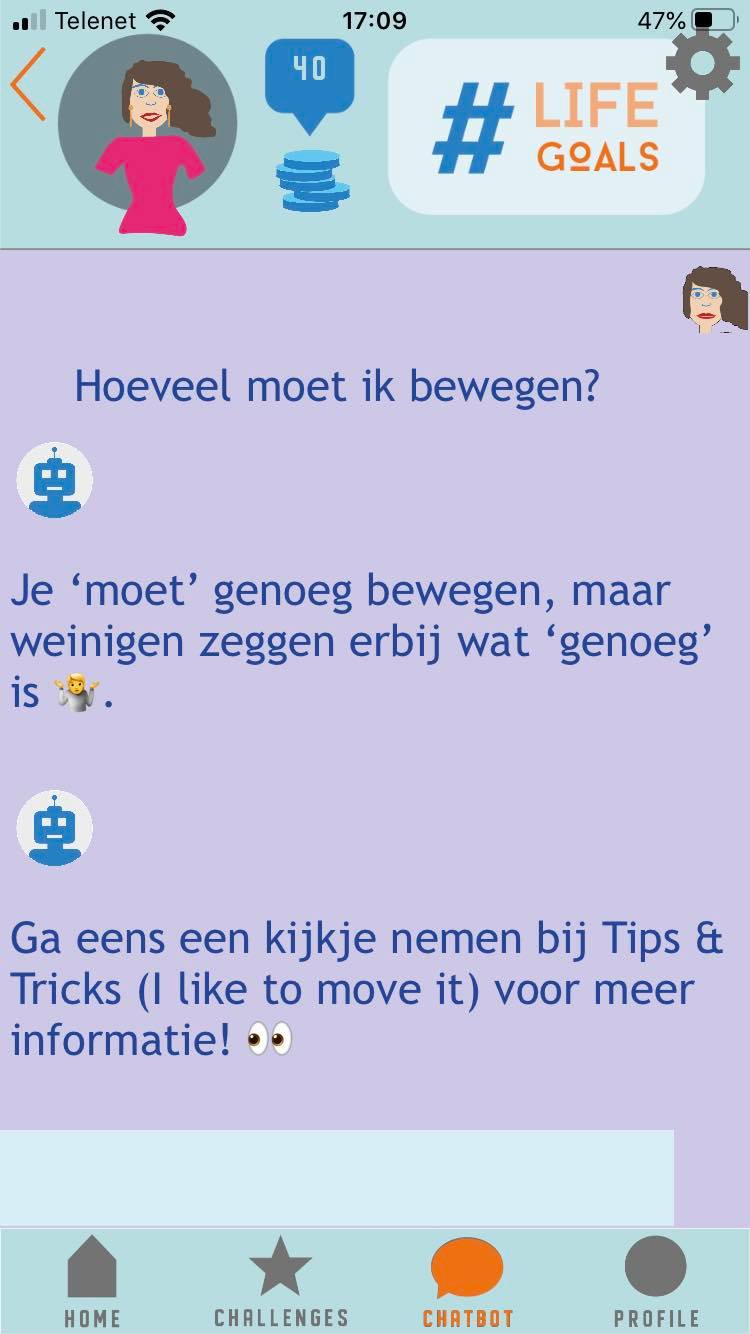


# Supplementary file 7

## Interview guide process evaluation

Then I have some questions about the chatbot. So about ‘Botje’ in the app.

*+ using prompt material (some images of the chatbot and the settings page)*

1. **What was it like to use the chatbot? *(FEASIBILITY)***

| Is the chatbot easy to use? Why yes/no? |
| --- |
| What did you like about the chatbot? |
| What did you dislike about the chatbot? |
| What did you think of the answers the chatbot gave to your questions? |
| What did you think of how the chatbot looked? What would you change and how? |
| Does the chatbot fit in the app? |

1. **Where did the chatbot help you with? *(THEORY OF CHANGE)***

| How did the chatbot help you with that? |
| --- |
| How did the chatbot support you in setting or reaching your missions? |

*If adolescents answer that the chatbot did not help them with anything:*

Maybe it didn't help you, but what do you think the intention of the chatbot was?

And why didn't it help you?

1. **When did you use the chatbot? *(CONTEXT)***

| Where were you? What were you doing? |
| --- |
| What prompted you to use the chatbot? |

*If they mention a situation/context in which they did not use the chatbot:*

What prevented you from using the chatbot?

# Supplementary file 8

## Overview of the social cues embedded in the chatbot

| **Category** | **Subcategory** | **Social cue** |
| --- | --- | --- |
| 1. **Verbal** | *Content* | (1) Use of humor and (2) empathy, (3) engage in small talk, (4) appropriate referrals, (5) express a name, (6) clear overall chatbot description by introducing itself to the user with a short statement, (7) adolescents are able to ask questions related to topics that are difficult to talk about with their parents, (8) ask for assistance with the app, (9) non-judgmental, (10) trustworthy |
|  | *Style* | (11) Short and precise interaction, (12) variation in system responses, (13) anonymity, (14) free dialogue, (15) avoidance of redundant answers, (16) youth language (i.e., informal style), (17) notifications, (18) accurate and realistic answers (19) formulated in a positive manner |
| 1. **Visual** | *Kinesics* |  |
|  | *Proxemics* |  |
|  | *Agent appearance* | (20) Profile picture, (21) personality, (22) cheerful design with (23) the ability to personalize |
|  | *Computer-mediated communication (CMC)* | (24) Emoticons |
| 1. **Auditory** | *Voice qualities* |  |
|  | *Vocalizations* |  |
| 1. **Invisible** | *Chronemics* | (25) Fast responses |
|  | *Haptics* |  |
